# Evaluation of carcinogenic modes of action for pesticides in fruit on the Swedish market using a text-mining tool

**DOI:** 10.3389/fphar.2014.00145

**Published:** 2014-06-23

**Authors:** Ilona Silins, Anna Korhonen, Ulla Stenius

**Affiliations:** ^1^Institute of Environmental Medicine, Karolinska InstitutetStockholm, Sweden; ^2^Computer Laboratory, University of CambridgeCambridge, UK

**Keywords:** pesticides, mode of action, text-mining, chemical carcinogenesis, chemical mixtures, risk assessment

## Abstract

Toxicity caused by chemical mixtures has emerged as a significant challenge for toxicologists and risk assessors. Information on individual chemicals' modes of action is an important part of the hazard identification step. In this study, an automatic text mining-based tool was employed as a method to identify the carcinogenic modes of action of pesticides frequently found in fruit on the Swedish market. The current available scientific literature on the 26 most common pesticides found in apples and oranges was evaluated. The literature was classified according to a taxonomy that specifies the main type of scientific evidence used for determining carcinogenic properties of chemicals. The publication profiles of many pesticides were similar, containing evidence for both genotoxic and non-genotoxic modes of action, including effects such as oxidative stress, chromosomal changes and cell proliferation. We also found that 18 of the 26 pesticides studied here had previously caused tumors in at least one animal species, findings which support the mode of action data. This study shows how a text-mining tool could be used to identify carcinogenic modes of action for a group of chemicals in large quantities of text. This strategy could support the risk assessment process of chemical mixtures.

## Introduction

Chemical risk assessment of mixtures is an important but challenging task for toxicologists. Unlimited variations of mixtures in our environment and knowledge gaps about toxic effects caused by chemical mixtures are examples of factors that make this process complex. Mixture effects can be described as caused either by additivity or interactions (such as synergistic or antagonistic effects) of the individual compounds (Feron and Groten, 2002). Strategies based on these theories have been developed to predict the toxic effects of mixtures, and include e.g., dose addition, which assumes a common or similar toxicological mode of action (MOA) of the individual chemicals (US EPA, 2000; Teuschler, 2007). The MOA of a substance can be defined as “the biologically plausible sequence of key events leading to an observed effect” (Sonich-Mullin et al., 2001; Boobis et al., 2006). Chemicals with the same or similar MOA may suggest potential for additive effects when such compounds are combined (US EPA, 2000).

Pesticides consist of a variety of chemicals with diverse mechanisms of action. Along with their plant protection characteristics these substances have intrinsic toxicological properties, which vary with the type of pesticide. Pesticide residues are frequently detected in analyses of fruits and vegetables and it is not unusual to detect more than one pesticide in the same fruit or vegetable. The European Food Safety Authority (EFSA) reports that the percentage of samples of fruits, vegetables and cereals with multiple residues increased by 11 percent from 1997 to 2007 (from 15 to 26 percent). In 2008, residues of two or more pesticides were found in 27 percent of the samples analyzed. The same proportion, 27 percent of samples containing multiple residues, was found in 2010. One sample of grapes was found to contain as many as 26 different pesticide residues (EFSA, 2010). As many fruits and vegetables contain more than one pesticide residue, theoretically, the combination of chemicals has the potential to cause mixture effects.

We have developed a text-mining tool, CRAB, for cancer risk assessment. CRAB classifies literature according to a taxonomy structurally based on currently established carcinogenic MOA (Korhonen et al., 2009, 2012; Sun et al., 2009). In this study we have used the CRAB tool to evaluate the carcinogenic MOAs reported in the literature for 26 pesticides found in apples and oranges on the Swedish market. More than 24 000 abstracts on these pesticides were retrieved from PubMed and were automatically classified by the tool. The literature concerning many of the 26 pesticides contained information both about genotoxic and non-genotoxic MOA, which may suggest potential risks for mixture effects. Furthermore, an evaluation of published cancer evidence showed that positive cancer findings have been found previously for the majority of these substances. This study demonstrates how an automatic text-mining tool could be used to identify carcinogenic MOAs for a group of chemicals in large quantities of textual data. This method could aid and support in the risk assessment process of mixtures.

## Material and methods

### Pesticide data

From a report published by the Swedish National Food Agency (NFA) (Jansson et al., 2011) the 15 most frequently detected pesticide residues in apples and oranges, respectively, were selected for a literature MOA analysis. The report summarizes the results from an analysis conducted within the Swedish national control programme coordinated by the EU [Commission Regulation (EC) No 1213/2008]. The analyses were performed by a Swedish accredited laboratory. Test results from the analysis of 1 105 different fruit and vegetable samples are presented in the report. The analysis covered a total of 316 different pesticide residues (Jansson et al., 2011). All apple and orange samples were analyzed as whole fruits and included the peel (personal communcation Anders Jansson Swedish NFA, 2012). As four pesticides were detected both in apples and oranges (carbendazim, chlorpyrifos, pyrimethanil, and thiabendazole) a total of 26 individual pesticides were included in the MOA evaluation (Table 1).

**Table 1:** **Literature data on the 26 most frequently detected pesticides in apples and in oranges on the Swedish market**.

**Pesticide^a^**	**Number of PubMed abstracts^b^**	**Number of MOA abstracts^c^**
**APPLES**		
Acetamiprid	230	51
Azinphosmethyl	253	49
Boscalid	51	14
Captan	514	146
Carbendazim	697	235
Chlorpyrifos	3056	672
Diphenylamine	1540	630
Indoxacarb	162	44
Iprodione	218	63
Phosmet	160	41
Pirimicarb	186	46
Pyraclostrobin	71	20
Pyrimethanil	119	24
Thiabendazole	2242	490
Thiachloprid	153	27
Total number of abstracts	9652	2552
Range	51–3056	14–672
**ORANGES**		
2-Phenylphenol	225	82
Carbendazim	697	235
Chlorpyrifos	3056	672
Cypermethrin	1536	500
Dicofol	298	41
Imazalil	258	73
Imidacloprid	1212	270
Lambda cyhalothrin	700	157
Malathion	2959	609
Methidathion	167	28
Prochloraz	203	86
Pyrimethanil	119	24
Pyriproxyfen	306	113
Trichlorophenol	794	155
Thiabendazole	2242	490
Total number of abstracts	14 772	3535
Range	119–3056	24–672

a Detected by The Swedish National Food Agency (Jansson et al., 2011).

b Total number of PubMed abstracts are shown.

c Abstracts classified as relevant for MOA analysis and distributed in the MOA taxonomy by the CRAB tool.

### Text mining-based mode of action analysis

We used a text mining-based tool as a method to analyze the published literature of the 26 pesticides. The purpose was to investigate the individual pesticides' MOAs. The literature from PubMed (http://www.ncbi.nlm.nih.gov/pubmed) was gathered via a search (in August 2013) using the names of the pesticides. A computational tool, CRAB, was used to analyze the abstracts. The tool classifies PubMed abstracts automatically according to a MOA taxonomy. The MOA taxonomy captures the current understanding of processes leading to carcinogenesis and is based on two main categories: genotoxic and non-genotoxic MOA. The taxonomy is further structured into sub-categories according to a classification of Hattis et al. (2009). A total of 25 sub-categories are currently included in the taxonomy. These 25 categories range from more common carcinogenic endpoints, such as mutations, to less studied effects such as aryl hydrocarbon receptor activation and peroxisome proliferation. In brief, the tool downloads all PubMed abstracts for a given chemical and automatically analyzes the abstracts according to the evidence which they provide for different carcinogenic MOAs in the abstract text. The tool generates a publication profile based on the literature data and displays the results in (mean) percent of the total number of abstracts for each chemical. By comparing the publication profiles of different substances shared properties of seemingly unrelated chemicals can be identified. CRAB is based on advanced text-mining technology and case studies have shown that the tool can obtain high accuracy MOA classification (Korhonen et al., 2009, 2012; Sun et al., 2009). Patterns found by classification of large amounts of textual data can also reveal hidden associations between different chemicals, such as similar MOAs shared by chemicals in mixtures. In addition, important data gaps could easily be identified. The development of the first version of the CRAB tool is presented in Korhonen et al. (2012). The tool is available at: http://omotesando-e.cl.cam.ac.uk/CRAB/request.html.

### Manual analysis of pubmed, IARC and US EPA cancer classifications

We investigated the literature of human and animal *in vivo* evidence of carcinogenicity for the 26 pesticides analyzed by the CRAB tool. A manual review of cancer evidence reported in the scientific literature for the selected pesticides was conducted using PubMed. Reports on increased numbers of tumors in animals or increased cancer risks in humans caused by named pesticides were regarded as positive evidence. Reports on pesticide mixtures with undefined compounds were not included, but no other exclusion criteria were used. The PubMed search was conducted in August 2013 using the names of the individual pesticides. Tumor types reported in animal experiments and from epidemiological studies (mainly exposed workers) are listed in Table 2. Cancer classifications of the individual pesticides from US-EPA and IARC were also reviewed (Table 2).

**Table 2:** **Tumor data reported in PubMed, cancer classifications by US-EPA and IARC for the pesticides most frequently detected in apples and oranges on the Swedish market 2009**.

**Pesticide**	**Tumor site^1^ (animal)**	**Tumor site^2^ (human)**	**Classification^3^**
2-Phenylphenol	Urinary bladder	–	a
Acetamiprid	–	–	a
Azinphosmethyl	Pancreas	–	a
Boscalid	Thyroid	–	b
Captan	Duodenum, liver, adrenal glands, kidney, uterus, small intestine	Breast	c,d
Carbendazim	Lymphomas	–	d,e
Chlorpyrifos	Lymphomas	Lung, rectal cancer, Hodgkin's lymphoma, glioma	f
Cypermethrin	Skin	–	e
Dicofol	Liver	Prostate, leukemia	d,e
Diphenylamine	(Liver foci)	Bladder cancer	a
Indoxacarb	–	–	a
Imazalil	Liver, thyroid	–	g
Imidacloprid	–	–	f
Iprodione	Liver, testicular, ovary	–	g
Lambda cyhalothrin	–	–	h
Malathion	Lung, liver, mammary glands	Breast, Non-Hodgkin's lymphoma, prostate	b,d
Methidathion	Liver	–	e
Phosmet	Liver, mammary gland	–	b
Pirimicarb	Lung, urinary bladder	–	g
Prochloraz	Liver	–	e
Pyraclostrobin	–	–	a
Pyrimethanil	–	–	e
Pyriproxyfen	–	–	f
Thiachloprid	Ovary	–	g
Trichlorophenol	Leukemia, liver	–	i
Thiabendazole	Urinary bladder, thyroid	–	c

1 Rats or mice experimental studies.

2 Data reported from studies on occupational exposures.

3 Classification according to US-EPA or IARC.

^a^Not likely to be carcinogenic to humans.

^b^Suggestive evidence of carcinogenicity, but not sufficient to assess human carcinogenic potential.

^c^Likely to be carcinogenic to humans following prolonged, high-level exposures, not likely to be a human carcinogen at dose levels that do not cause cytotoxicity and regenerative cell hyperplasia.

^d^Classified by IARC as group 3 carcinogen (not classifiable as to its carcinogenicity to humans).

^e^Group C: possible human carcinogen.

^f^Group E:evidence of non-carcinogenicity for humans.

^g^Likely to be carcinogenic to humans.

^h^Group D: not classifiable as to human carcinogenicity.

^i^group B2: Probable human carcinogen.

## Results

Data from an analysis of apple and orange samples carried out in 2009–2010 was kindly provided by the Swedish NFA. Data shows that the majority of the tested apples and oranges contained several pesticide residues, 78 percent of the apple samples contained more than one pesticide and for orange samples this number was as high as 96 percent. The results thus show that two or more pesticide residues were freqently detected in apple and orange samples. For example, one apple sample contained residues of seven pesticides and in one orange sample 10 different pesticide residues were detected.

### Text mining-based mode of action analysis

**Figure 1:**
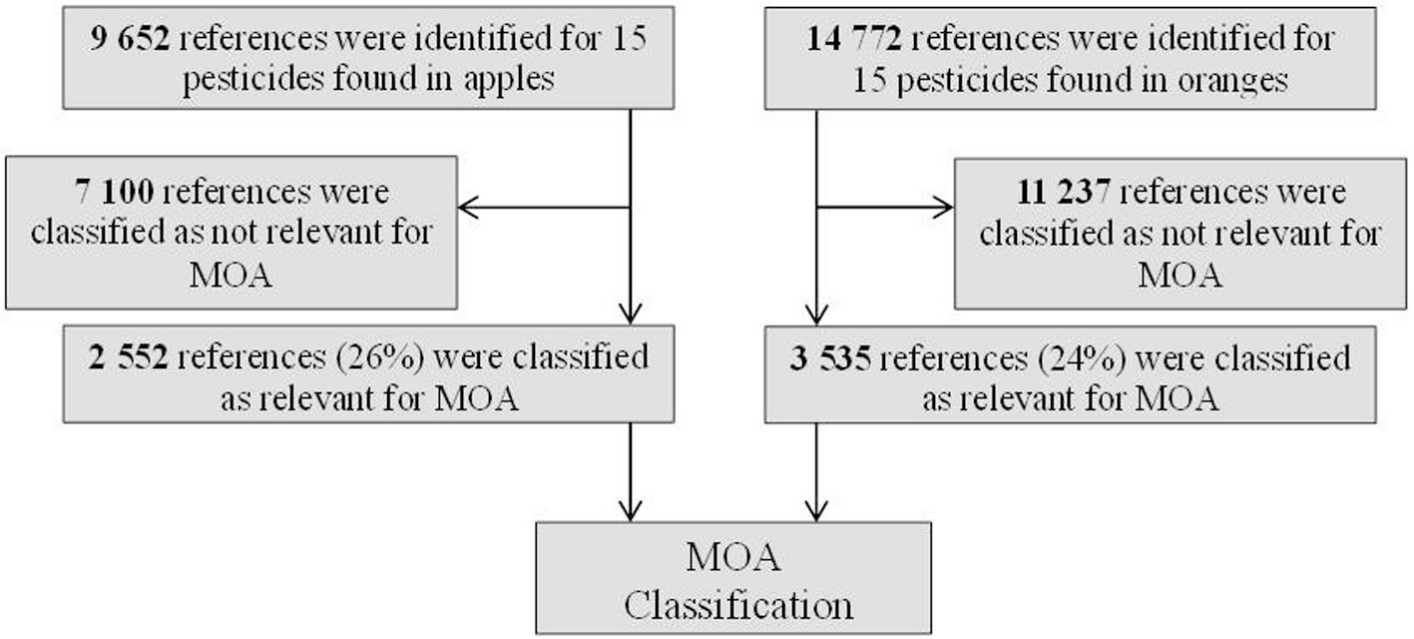
**Schematic flow chart of the tool used for classifying abstracts on the 26 selected pesticides**.

Table 1 shows the 15 most frequently detected pesticides/residues in apples and the 15 most frequently detected residues in oranges from the analysis of the Swedish National Food Agency (NFA) (Jansson et al., 2011). The total number of published PubMed abstracts concerning the 15 pesticides detected in oranges was higher compared to the apple pesticides (14 772 and 9 652 abstracts respectively). To conduct a MOA analysis of these pesticides we used the CRAB tool to analyze the literature. For each pesticide the tool classified the published abstracts automatically according to the MOA taxonomy. In Figure 1, a schematic flow chart of the classification of abstracts is shown. The tool identified 2 552 and 3 535 abstracts, respectively, as relevant for MOA classification and classified 18 337 as irrelevant (Figure 1). Thus, only 25 percent of the original PubMed collection was classified by the tool as relevant for cancer MOA classification and 75 percent of the retrieved articles were deemed by the tool not to be relevant for MOA, requiring no further examination. Based on the results from the MOA classification, the group of orange pesticides was in general studied more widely than the group of apple pesticides (Table 1). The range of abstracts showed that some pesticides were less studied (e.g., only 14 boscalid abstracts were relevant for MOA), while other pesticides were more well-studied (e.g., chlorpyrifos and malathion, 672 and 609 abstracts, respectively). The information about data gaps may also be important and could point to knowledge gaps that require more research.

**Figure 2:**
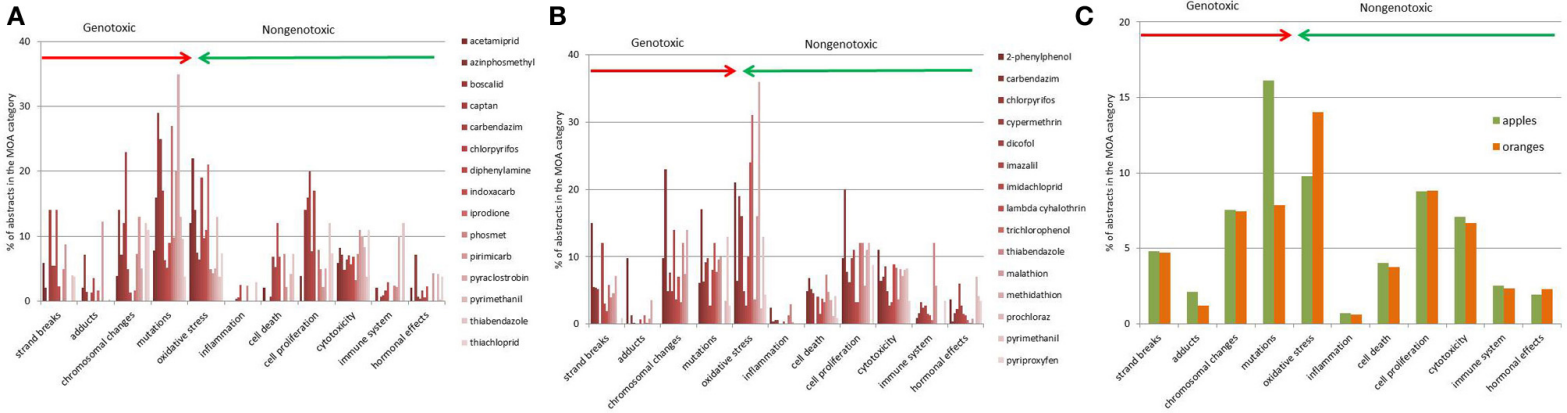
**Literature distribution of the 15 individual pesticides most commonly detected in apple samples, data is shown for 11 selected sub-categories (A)**. The literature distribution over the MOA taxonomy for individual pesticides most commonly detected in oranges, data is shown for 11 selected sub-categories **(B)**. The literature distribution of 15 pesticides commonly detected in apples and 15 pesticides commonly found in oranges (shown as the mean percentage of abstracts found in the MOA category). Data is shown for 11 selected sub-categories **(C)**.

The distribution of abstracts over the MOA taxonomy was analyzed in detail. The distribution of classified abstracts for the individual pesticides in apples and oranges is shown for 11 selected common MOA categories (Figures 2A,B). The distribution pattern suggests that most pesticide abstracts were assigned to the mutation and oxidative stress category. Other frequent categories were chromosomal changes, cell proliferation and cytotoxicity. However, a detailed examination of abstracts in the mutation category for both apple and orange pesticides revealed that part of the literature was related to resistance-linked mutations in crops and not particularly to the mutagenic potential of the pesticides. The combination of MOA profiles for both apple and orange pesticides is shown in Figure 2C (which is based on the same data as in Figures 2A,B). In summary, the profiles indicate close similarities, e.g., for both apple and orange pesticides much information concerning oxidative stress, chromosomal changes and cytotoxicity was found.

### Cancer classifications and published data of pesticides

We compared the MOA analysis by the CRAB tool with carcinogenic evidence and classifications for each pesticide (Table 2). Common tumor sites reported in animals studies were liver, urinary bladder, mammary gland, thyroid and the lymphatic system. Tumor types reported from human studies (mainly exposed workers) were lung, breast and prostate tumors, non-Hodgkin's lymphoma and rectal cancer. We further examined the US-EPA IRIS cancer classifications [Integrated risk information system (IRIS) 2012]. This analysis showed that among the 26 pesticides studied here six were classified as “possible human carcinogen,” four as “likely to be carcinogenic to humans following prolonged, high-level exposures, not likely to be a human carcinogen at dose levels that do not cause cytotoxicity and regenerative cell hyperplasia,” three pesticides were classified with “suggestive evidence of carcinogenicity, but not sufficient to assess human carcinogenic potential” and one pesticide as “probably human carcinogen.” According to the US-EPA/IRIS evaluations, four pesticides were not classifiable because of insufficient data; three pesticides were classified as “evidence of non-carcinogenicity for humans” and six pesticides as “not likely to be carcinogenic to humans.”

When all information on cancer classifications and published studies of the 26 pesticides was summed, it showed that the majority of the chemicals have evidence or classifications that suggest carcinogenic potential. The animal tumor data retrieved from published literature through PubMed shows evidence of carcinogenicity reported previously for 18 of 26 substances (Table 2). Thus, published data show that the majority of the 26 compounds in apples and oranges on the Swedish market have published evidence of carcinogenic properties, and have potential to function either as tumor promoters or initiators. This is in line with the MOA classification findings showing genotoxic and non-genotoxic effects for the majority of the 26 pesticides.

## Discussion

In this study we used an automatic text mining-based tool for literature review and MOA analysis to evaluate 26 pesticides that were frequently found in apples and oranges on the Swedish market. The results obtained in this study indicate that pesticides commonly detected in these fruits may possess similar genotoxic and non-genotoxic properties.

The literature profiles generated by the text-mining tool showed that genotoxic MOA categories, such as chromosomal changes and strand breaks, and indirect/non-genotoxic MOA, such as oxidative stress, were common for pesticides detected in apples and oranges. Regarding data on chromosomal damages and oxidative stress, these endpoints have previously demonstrated to be associated with pesticide exposure and cancer development (Giri et al., 2002; Abdollahi et al., 2004; Mena et al., 2009). For example, several studies of workers exposed to pesticide mixtures have reported increased levels of chromosomal aberrations, sister chromatid exchanges and micronuclei (Bolognesi, 2003). One study reports on high levels of chromosomal abnormalities that are linked to an increased risk of Non-Hodgkin's lymphoma in workers exposed to pesticides (Chiu and Blair, 2009).

We found that the literature on many pesticides contained non-genotoxic data, including cell proliferation, cytotoxicity and effects on the immune system. This is in line with previous studies reporting on carcinogenic pesticides as tumor promoters (Rakitsky et al., 2000). Pesticides are designed to be toxic, and regenerative cell proliferation might for example be a result of cytotoxicity. Some pesticides are considered to have endocrine disrupting properties (Choi et al., 2004) and among the substances included in this study and reported to have endocrine disrupting effects are captan, carbendazim, chlorpyrifos, cypermethrin, dicofol, iprodione, malathion, prochloraz, and pyriproxyfen (Committee on Toxicity, 2002; Mnif et al., 2011). In summary, we found that many of the pesticide residues in apples and oranges have similar publication profiles, as generated by the CRAB tool. Similarities may reflect shared MOA, which in turn may result in additive or interaction effects when these compounds are combined.

Risk assessment of pesticide residues in fruit and vegetables is a complicated matter and there is a concern about the effect of pesticides residue mixtures in the diet. To protect people from harmful effects of pesticides the European Commission has set maximum residue levels (MRL) for all pesticides permitted in food products intended for human consumption (EFSA, 2012). Individual MRL values are set so that the pesticide intake does not exceed the ADI (acceptable daily intake) of the individual pesticides (Renwick, 2002), but these are developed for individual compounds and do not take into account potential mixture effects of combined pesticides. Among the samples analyzed by the Swedish NFA in 2009–2010 we found that almost all of the orange samples, as well as the majority of the apple samples, contained more than one pesticide residue. This means eating just one fruit is likely to expose you to at least a binary mixture of pesticides. Although the individual pesticide residues in fruit are usually found at low concentrations combined exposure may result in mixture effects if these compounds share similar MOAs. Another factor that complicates human risk assessment of pesticide mixtures is that very little data on human internal doses and blood levels for exposure analysis is available for many pesticides, which makes it difficult to estimate human exposure.

The CRAB tool has many advantages over manual literature analysis when large quantities of data need to be examined. The tool provides a rapid view of published literature and can point to carcinogenic MOA that groups of chemicals can have in common. We have previously conducted case studies to demonstrate how the text-mining tool can be used to support cancer risk assessment. For example, literature profiles of well-known carcinogens were compared with the known properties of each chemical and the classification results correlated well to what was previously known about these substances (Korhonen et al., 2012). The tool has also been used for hypothesis generation (Kadekar et al., 2012). To compare groups of chemicals using a text-mining tool may be very helpful to risk assessors and researchers, as common associations between seemingly different chemicals can be difficult to detect by manual means. In this study, 24 424 abstracts on pesticides were found in the original PubMed search, from which the tool classified 25 percent (6 087 abstracts) as relevant to carcinogenic MOAs. The working load of evaluating all abstracts manually was thus reduced with 75 percent. Regarding risk assessment of mixtures, a MOA shared by several chemicals can indicate potential to cause mixtures effects. The classified data could generate hypotheses of common MOA that could be further tested experimentally. While data- and text mining-based approaches could be highly useful for researchers and risk assessors to support scientific evaluation the current tool has also some limitations. The method is primarily intended as a first step to support risk assessors and researchers in the initial phase of the hazard identification step. Although the tool provides literature profiles that may suggest potential associations between chemicals, for conducting complete hazard identification the retrieved articles will always need to be examined further in detail. Additionally, because pesticides may act by more than one MOA, which can be both cell- and dose-specific, the classification results may require a more detailed investigation on dose-response and tissue-specific effects. Additional efforts for risk assessment would include detailed exposure assessments and in some cases further experimental work to confirm results. To support scientific evaluation based on text on a more detail level, we have plans to develop methods for automatic extraction of data from articles. Recently, a reader function was incorporated into the tool that enables automatic analysis of the abstract information structure. This function could be used to further improve the tool and promote scientific conclusion.

Hazard identification and risk assessment of mixtures is a complex and challenging process. The study described here provides an example of how a text mining-based tool could support the analysis of large amounts of textual information to detect trends and patterns in data. A MOA analysis can identify common links between different chemicals which could serve as basis for hypothesis generation and direct further research and risk assessment of chemical mixtures.

## Author contributions

All authors were involved in the design of the study. Ilona Silins conducted the text mining experiments, analysis and the manual literature review. All authors contributed to the writing of the manuscript. All authors approved the final manuscript.

### Conflict of interest statement

The authors declare that the research was conducted in the absence of any commercial or financial relationships that could be construed as a potential conflict of interest.
